# Open versus minimally invasive TLIF: literature review and meta-analysis

**DOI:** 10.1186/s13018-019-1266-y

**Published:** 2019-07-22

**Authors:** Ahmed Hammad, André Wirries, Ardavan Ardeshiri, Olexandr Nikiforov, Florian Geiger

**Affiliations:** Spine Centre, Hessing Foundation, Hessingstrasse 17, 86199 Augsburg, Germany

**Keywords:** Lumbar, Interbody fusion, Transforaminal, Open, Minimally invasive

## Abstract

**Study design:**

This study is a comparative, literature review.

**Objective:**

The aim of this study is to provide a comparative analysis of open vs. minimally invasive TLIF using a literature review and a meta-analysis.

**Summary of background data:**

Lumbar interbody fusion is a well-established surgical procedure for treating several spinal disorders. Transforaminal lumbar interbody fusion (TLIF) was initially introduced in the early 1980s. To reduce approach-related morbidity associated with traditional open TLIF (OTLIF), minimally invasive TLIF (MITLIF) was developed. We aimed to provide a comparative analysis of open vs. minimally invasive TLIF using a literature review.

**Methods:**

We searched the online database PubMed (2005–2017), which yielded an initial 194 studies. We first searched the articles’ abstracts. Based on our inclusion criteria, we excluded 162 studies and included 32 studies: 18 prospective, 13 retrospective, and a single randomized controlled trial. Operative time, blood loss, length of hospital stay, radiation exposure time, complication rate, and pain scores (visual analogue scale, Oswestry Disability Index) for both techniques were recorded and presented as means. We then performed a meta-analysis.

**Results:**

The meta-analysis for all outcomes showed reduced blood loss (*P* < 0.00001) and length of hospital stay (*P* < 0.00001) for MITLIF compared with OTLIF, but with increased radiation exposure time with MITLIF (*P* < 0.00001). There was no significant difference in operative time between techniques (*P* = 0.78). The complication rate was lower with MITLIF (11.3%) vs. OTLIF (14.2%), but not statistically significantly different (*P* = 0.05). No significant differences were found in visual analogue scores (back and leg) and Oswestry Disability Index scores between techniques, at the final follow-up.

**Conclusion:**

MITLIF and OTLIF provide equivalent long-term clinical outcomes. MITLIF had less tissue injury, blood loss, and length of hospital stay. MITLIF is also a safe alternative in obese patients and, in experienced hands, can also be used safely in select cases of spondylodiscitis even with epidural abscess. MITLIF is also a cost-saving procedure associated with reduced hospital and social costs. Long-term studies are required to better evaluate controversial items such as operative time.

## Introduction

Lumbar interbody fusion (LIF) is a well-established surgical procedure used to treat several spinal disorders including degenerative disease, trauma, infection, and neoplasia [1]. The procedure involves placing an implant (spacer, graft, or cage) within the intervertebral space after discectomy [2]. Transforaminal lumbar interbody fusion (TLIF), a spinal fusion posterior approach, was initially described by Harms and Rollinger in 1982 [3], and gained popularity after work by Harms and Jeszenszky in 1998 [4]. This technique achieves 360° of circumferential fusion via a single posterolateral approach [3] with less retraction, and thus reduced risk, to the central neural structures, namely the dura. The main disadvantages of TLIF include significant muscle retraction and dissection. Minimally invasive TLIF (MITLIF) was introduced to minimize iatrogenic soft tissue and muscle injury associated with traditional open TLIF (OTLIF) while maintaining comparable clinical, radiological, and economic outcomes. This is a novel surgical procedure first described in the early 2000s by Foley et al. [5] that involves serially inserting tubular retractors via a muscle-dilating approach, which minimizes iatrogenic muscle injury [6].

### History

#### TLIF

TLIF was first reported in the early 1980s as a modification of posterior LIF. As a newer technique, TLIF creates circumferential fusion via a more lateralized unilateral posterolateral approach, without the need to expose the contralateral foramen. This approach reduces retraction of the central neural structures and minimizes the risk of central neurological injury [3].

#### MITLIF

Developments in instrumentation and imaging have increased the trend and evolution toward minimally invasive spinal surgery [7]. To minimize tissue trauma associated with traditional open posterior approaches, percutaneous pedicle screw-rod fixation systems were developed [7]. These were initially reported by Foley and colleagues in 2001 for posterior LIF [8] and also by other researchers for TLIF [9, 10]. MITLIF was first described by Foley et al. in 2002 [5], who used sequential dilation, tubular retraction, and a percutaneous screw-rod system. MITLIF has since gained popularity and has been used to treat various spinal diseases requiring fusion [11, 12].

### Technique

There are certain technical differences between MITLIF and OTLIF. First, in MITLF, intraoperative fluoroscopy is performed to locate the desired spinal level, and the pedicle screws are placed percutaneously above and below the desired interbody fusion segment. Then, a 1–2-in paramedian incision is made on the lateral borders of the facet joints of the desired spinal level on the side of the radicular symptoms. Serial soft tissue dilators are then inserted down to the facet complex. This is followed by spinal canal decompression, disc preparation, and cage insertion in a similar manner as for OTLIF. The tubular retractor is then removed, and finally, compression is applied to the rods before final tightening [13].

## Materials and methods

### Search method and inclusion criteria

**Fig. 1:**
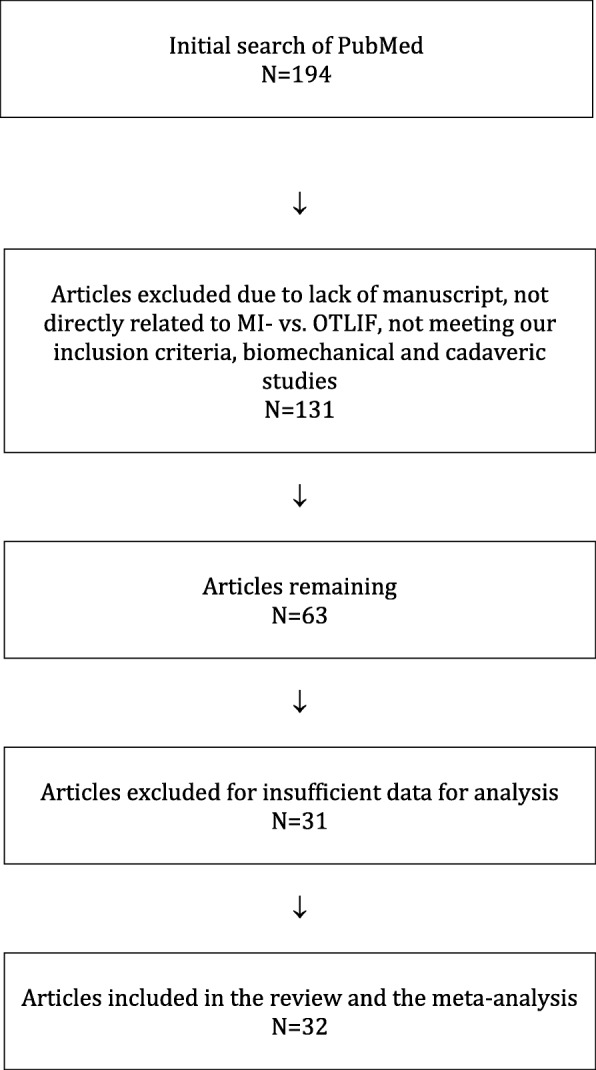
Flow diagram of the search strategy and study selection process

We reviewed the online database PubMed from 2005 to 2017 using the keywords ‘lumbar,’ ‘interbody fusion,’ ‘transforaminal,’ ‘open,’ and ‘minimally invasive,’ which identified 194 studies. We first screened all articles by their abstracts, and we included only English language reports with full text manuscripts. Additional inclusion criteria included (1) studies with a comparative design, (2) studies with populations consisting of adult patients > 18 years of age treated with MITLIF, (3) studies including a control group of patients treated with OTLIF, and (4) studies comparing at least one desirable outcome (e.g., operative time, blood loss, recovery time, or costs). Biomechanical and cadaveric studies and reviews were excluded. After applying our inclusion criteria, 162 studies were excluded leaving 32 articles for inclusion and review (Fig. 1).

### Statistical methods for the meta-analysis

We analyzed data from the included studies using Review Manager (RevMan version 5.2, The Nordic Cochrane Center, The Cochrane Collaboration, Copenhagen, Denmark), and Microsoft Excel 2010 (Microsoft Corp., Redmond, WA, USA). A formal meta-analysis was conducted for all outcomes if the data were sufficient. We expressed pooled dichotomous data as odds ratio (OR) with 95% confidence intervals (95% confidence interval (CI)); while pooled continuous effect measures was expressed as the mean difference with 95% CI. We explored and quantified between-study statistical heterogeneity using the *I*^2^ test. By default, we used the fixed-effect model in all analyses. If heterogeneity was statistically significant (*p* < 0.05) or *I*^2^ was > 50%, we used the Der Simonian and Laird random-effects model instead [43]. Statistical analyses were two-sided with an α-error of 0.05.

### Description and interpretation of the forest plots

For quantitative data, the forest plots are composed of (from left to right):

1. The names of the studies arranged by publication year.
2. Data for treatment and control groups including mean, SD, and number of patients in each group.
3. The weight of each study as a percentage of the total of the meta-analysis (100%).
4. The mean difference between the two groups + 95% CI.
5. Year of publication again.
6. On the right side is a figure depicting the results. Each study is represented by a square (square size = study weight, and the square’s center is opposite to the mean’s difference) on a straight line representing the 95% CI of the mean’s difference. The final results of the meta-analysis are represented by a black diamond and the center of the diamond is the mean’s difference across all studies. The tips of the diamond are the 95% CI of the mean’s difference across all studies.
7. The line in the middle of the graph is opposite the 0 value and termed the equator line, which means no difference between the groups. If the lines of any study and/or the diamond touch the equator line, there is a statistical difference between the two groups.
8. The last two lines written in the plot indicate the heterogeneity represented by the *I*^2^ statistic as a % and a *p* value. With *p* < 0.05, heterogeneity is considerable across the studies, and the results should be considered cautiously. The second line is the *p* value of the overall results, represented by the diamond in the graph. These results are represented by Z and *p* values. When *p* is < 0.05, the overall result is statistically significant.

For qualitative data, the forest plots are composed of (from left to right):

1. The names of the studies arranged by publication year.
2. Data for treatment and control groups including the number of positive events and the total number of patients in each group.
3. The weight of each study as a percentage of the total of the meta-analysis (100%).
4. The OR between the two groups with 95% CI.
5. The publication year again.
6. On the right side, there is a figure depicting the results. Each study is represented by a square with the size of the square = study weight, and the center of the square opposite the OR, on a straight line representing the 95% CI. The result of the meta-analysis is represented by the black diamond with the center of the diamond indicating the OR across all studies and the tips indicating the 95% CI across all studies.
7. The line in the middle of the graph is opposite the 1 value and termed the equator line, which means no difference between the groups. If the lines of any study and/or the diamond touch the equator line, there is statistical difference between the two groups.
8. The last two lines of the plot indicate heterogeneity represented by the *I*^2^ statistic as a percentage and a *p* value. When the *p* value is < 0.05, heterogeneity is considerable across the studies, and the results should be interpreted cautiously. The second line is the *p* value of the overall results (represented by the diamond in the graph). The overall results are represented by *Z* and *p* values. When *p* is < 0.05, the overall result is statistically significant.

## Results

### Search results

**Fig. 2:**
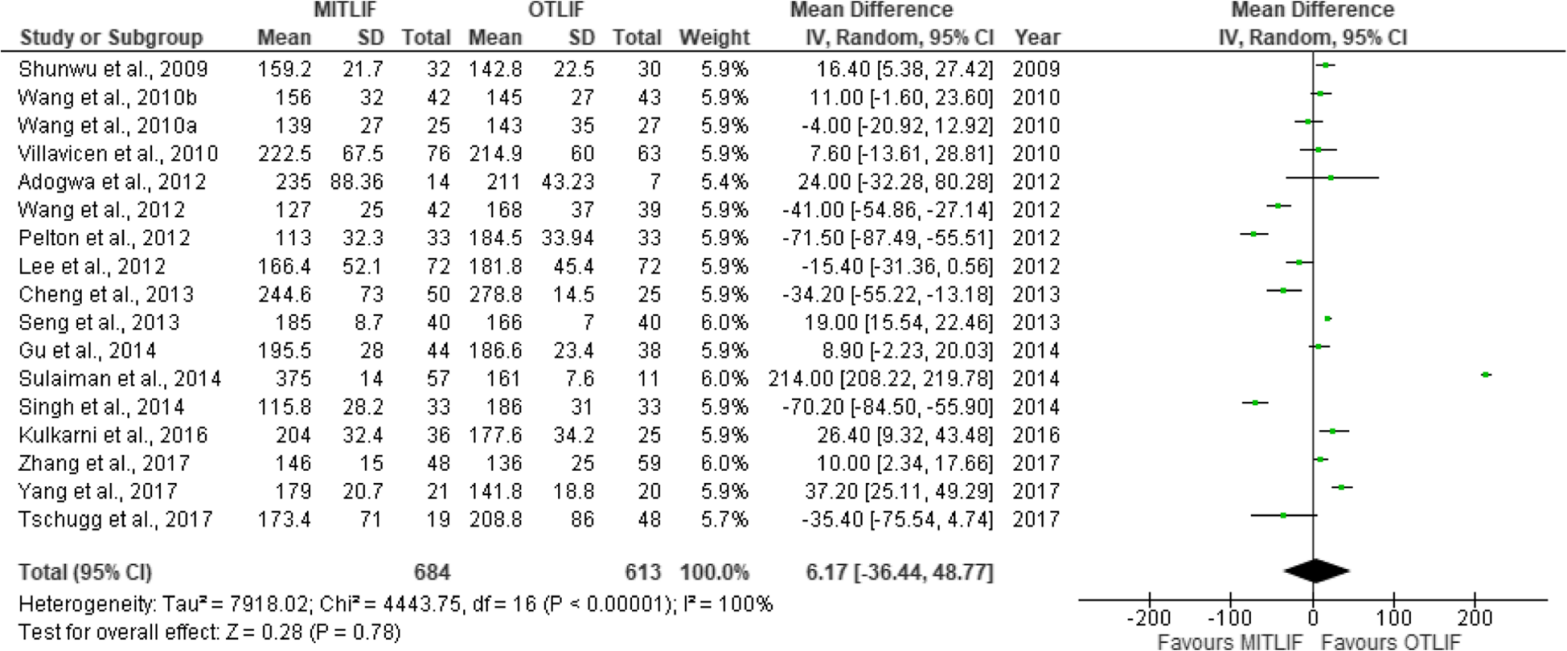
Forest plot of the study comparisons: comparison between MITLIF and OTLIF outcomes for operative time

**Fig. 3:**
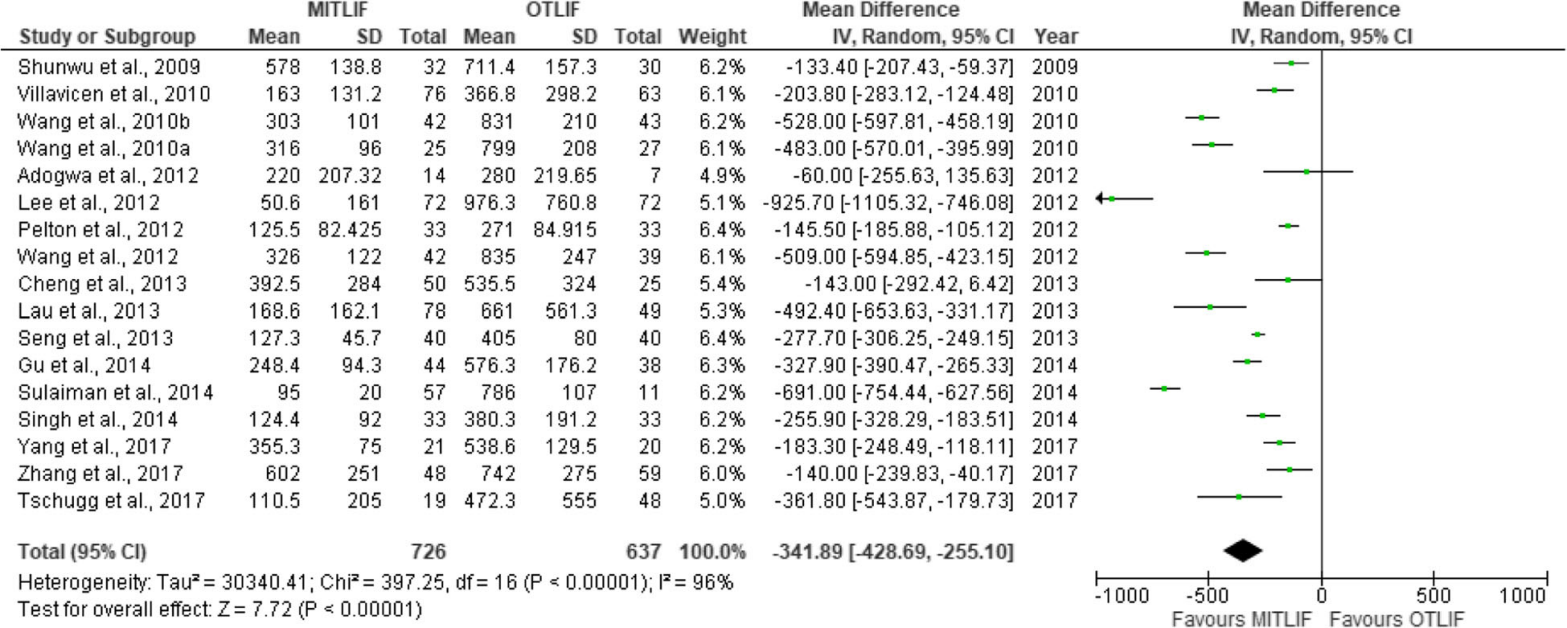
Forest plot of the study comparisons: comparison between MITLIF and OTLIF outcomes for blood loss

**Fig. 4:**
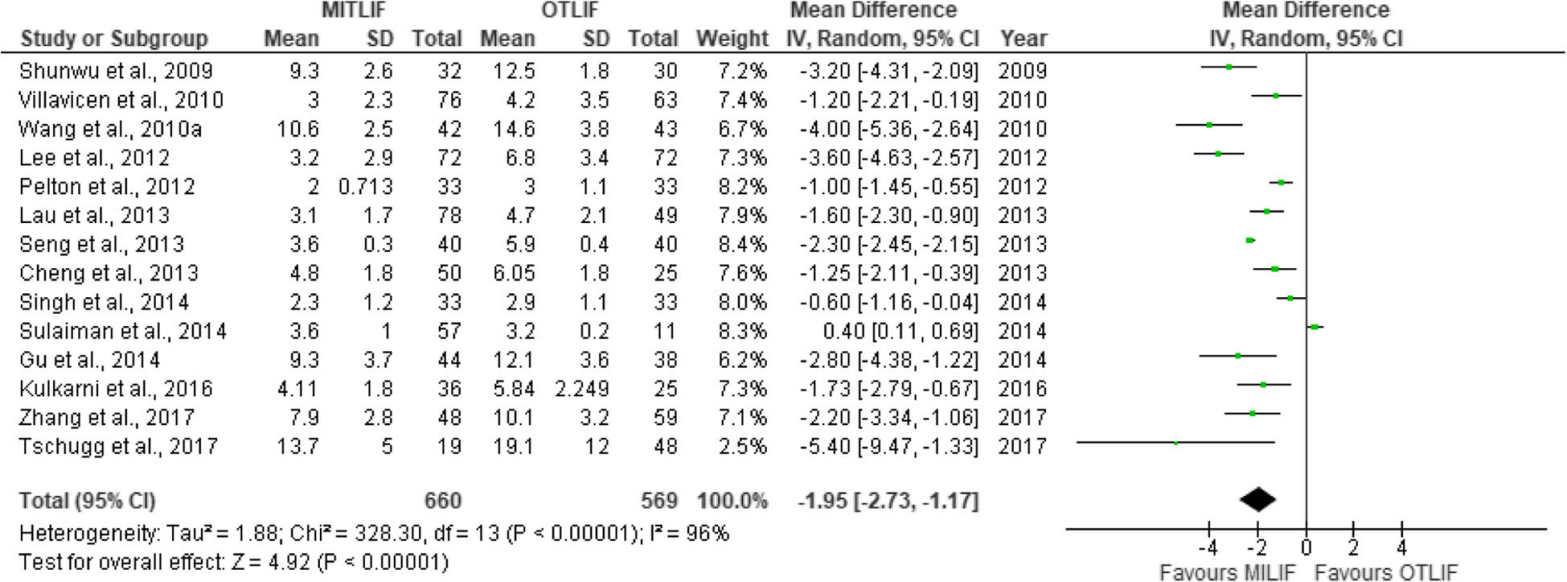
Forest plot of the study comparisons: comparison between MITLIF and OTLIF outcomes for length of hospital stay

**Fig. 5:**
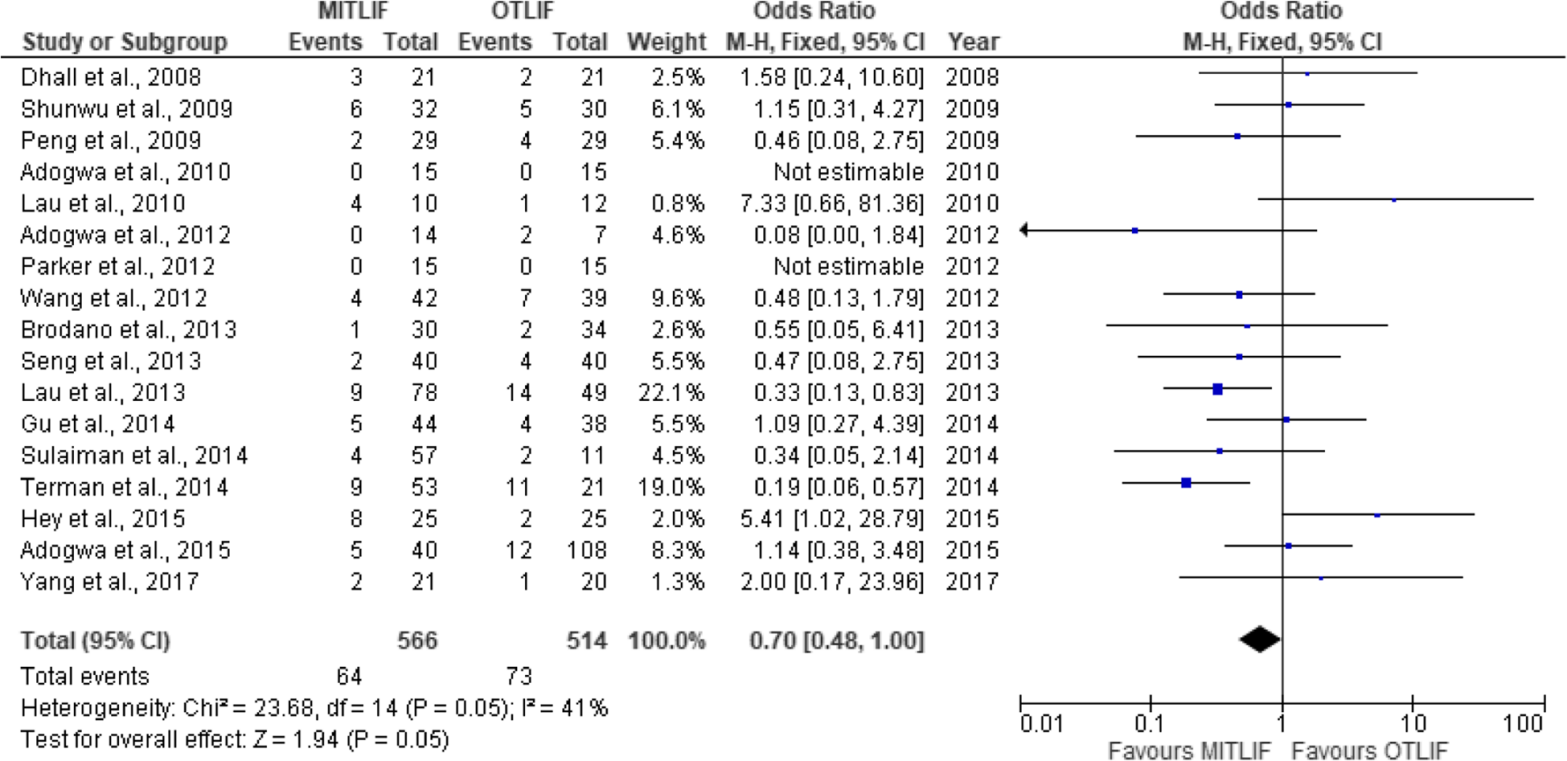
Forest plot of the study comparisons: comparison between MITLIF and OTLIF outcomes for: complications

**Fig. 6:**
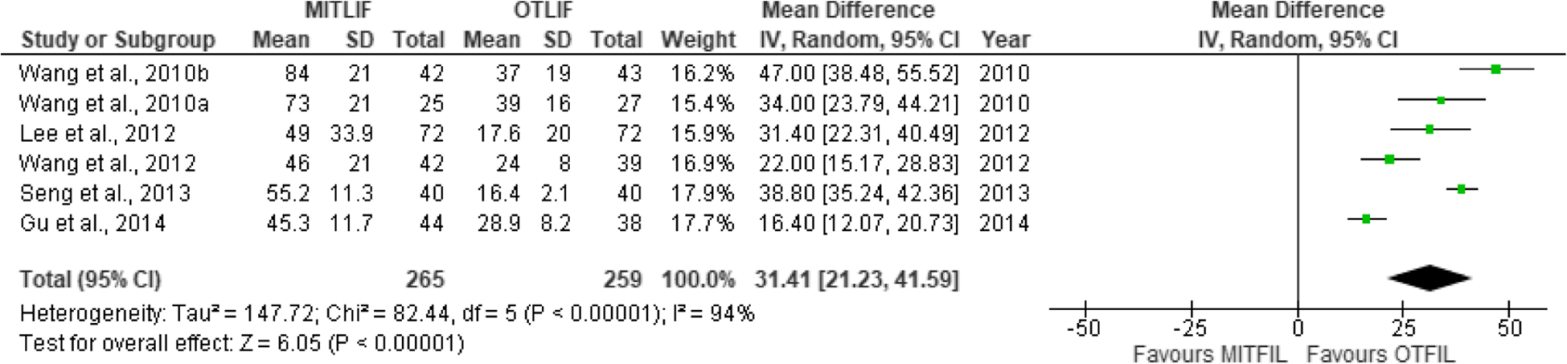
Forest plot of the study comparisons: comparison between MITLIF and OTLIF outcomes for radiation exposure time

**Fig. 7:**
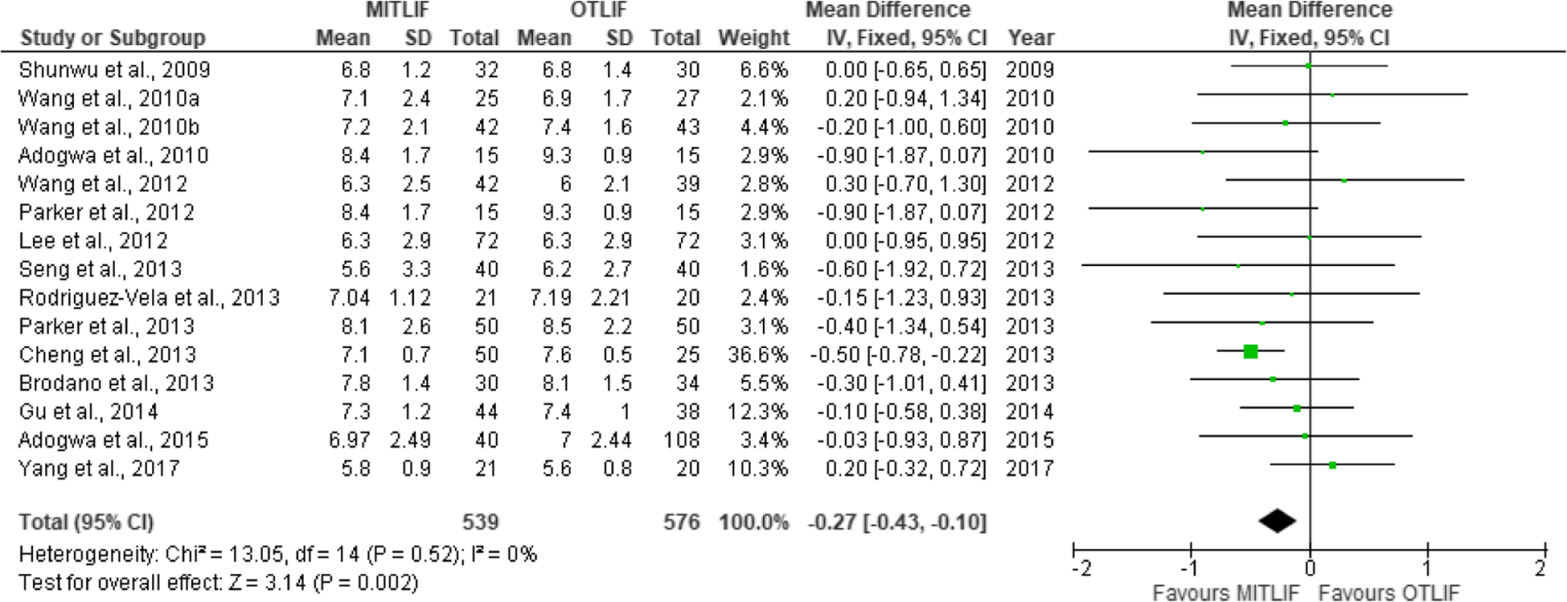
Forest plot of the study comparisons: comparison between MITLIF and OTLIF outcomes for preoperative visual analog scale scores for back pain

**Fig. 8:**
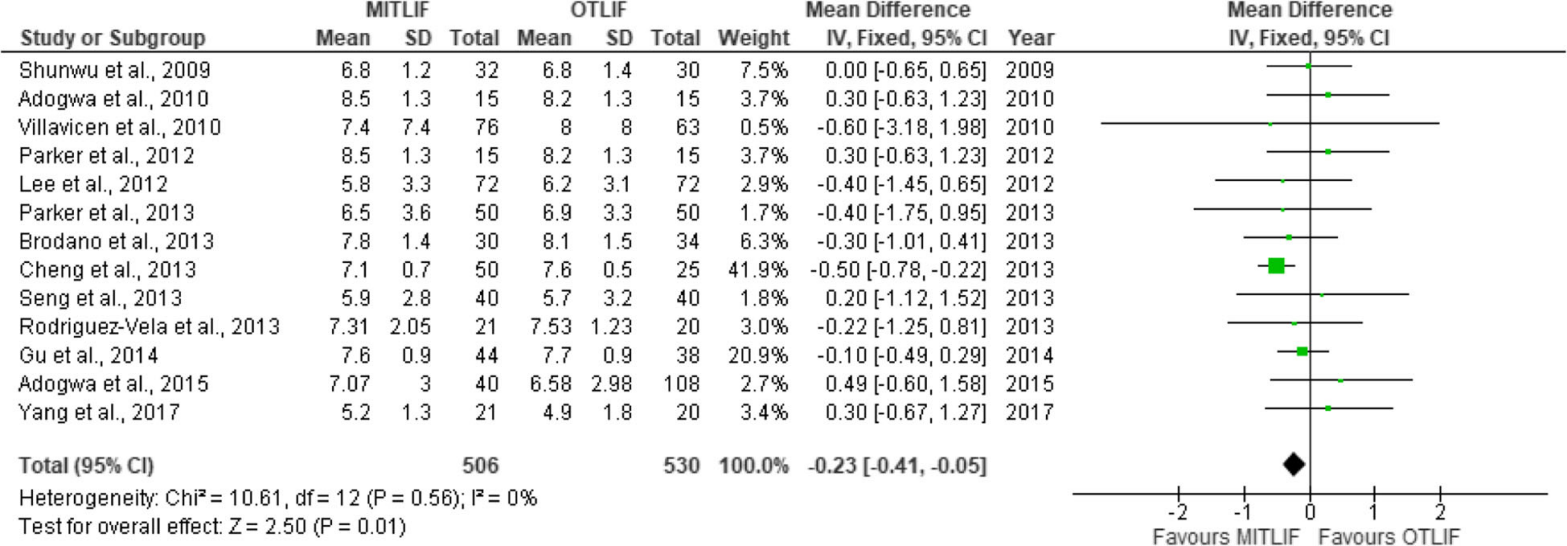
Forest plot of the study comparisons: comparison between MITLIF and OTLIF outcomes for preoperative visual analog scale scores for leg pain

**Fig. 9:**
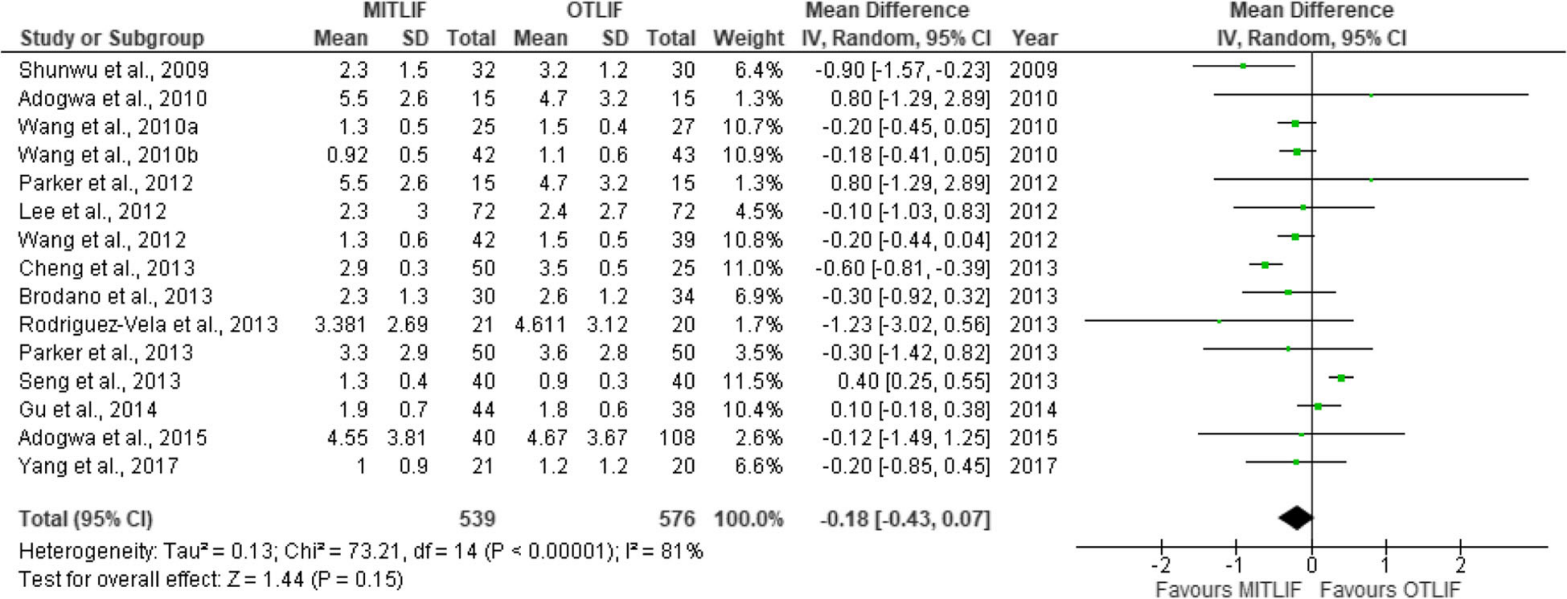
Forest plot of the study comparisons: comparison between MITLIF and OTLIF outcomes for visual analog scale scores for back pain at final follow-up

**Fig. 10:**
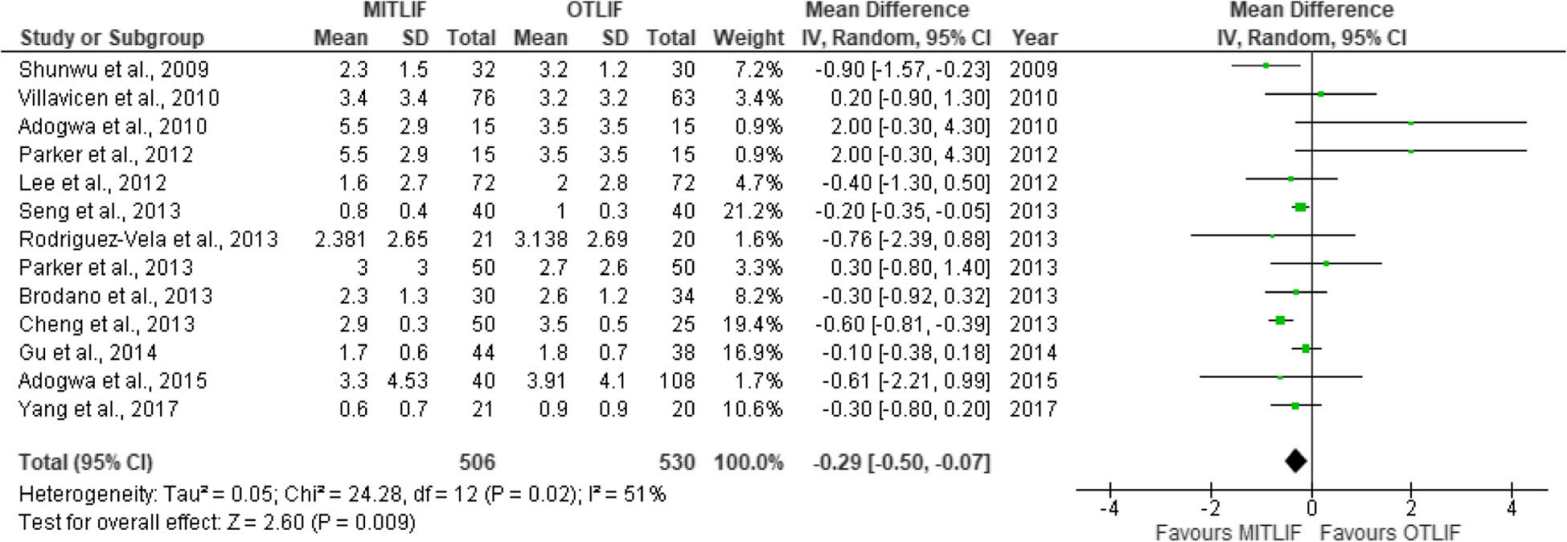
Forest plot of study comparisons: comparison between MITLIF and OTLIF outcomes for visual analog scale scores for leg pain at last follow-up

**Fig. 11:**
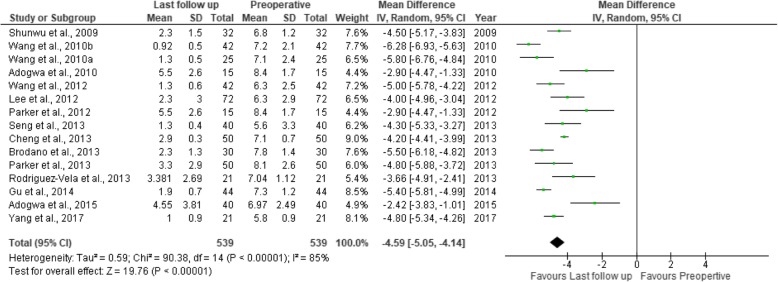
Forest plot of study comparisons: comparison between preoperative and last follow-up visual analog scale scores for back pain within the MITLIF group

**Fig. 12:**
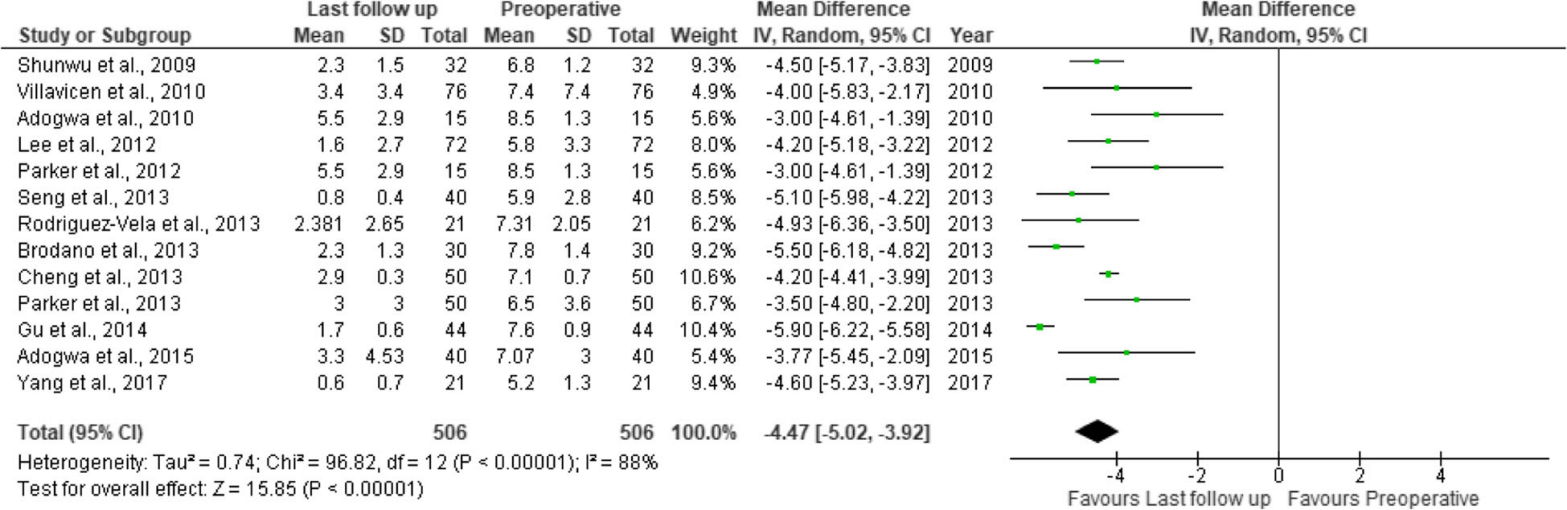
Forest plot of study comparisons: comparison between preoperative and last follow-up visual analog scale scores for leg pain within the MITLIF group

**Fig. 13:**
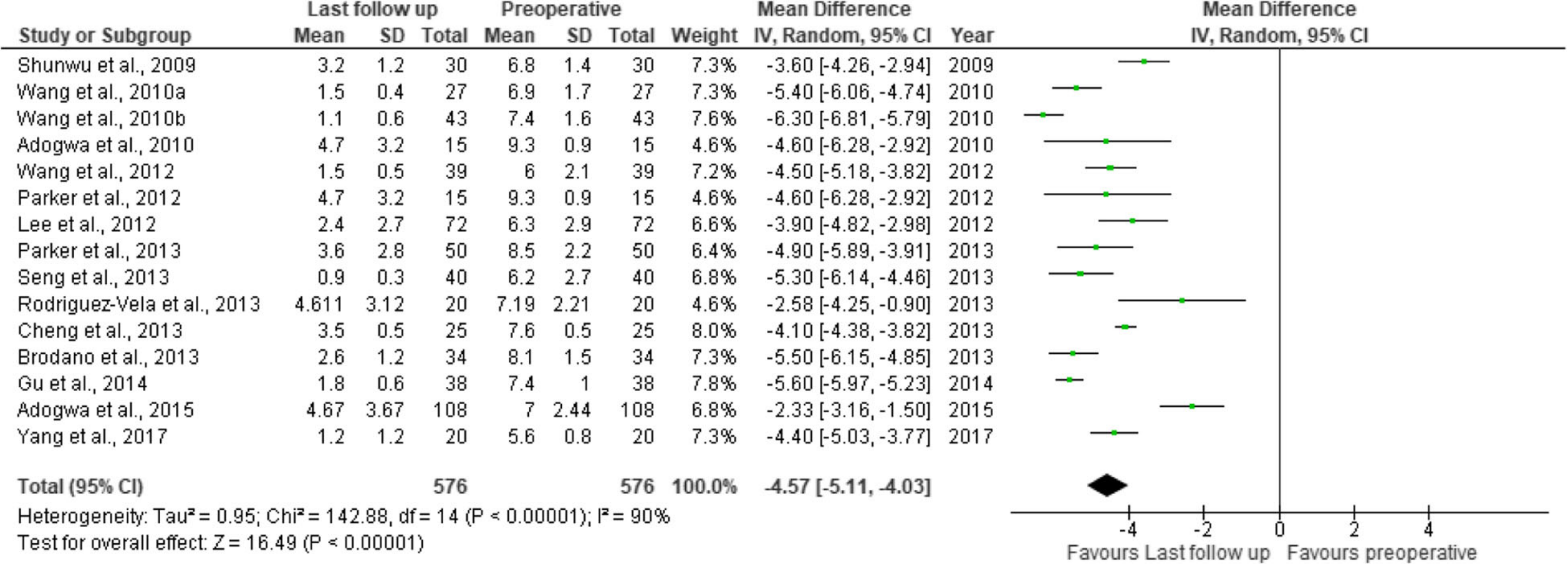
Forest plot of the study comparisons: comparison between preoperative and last follow-up visual analog scale scores for back pain within the OTLIF group

**Fig. 14:**
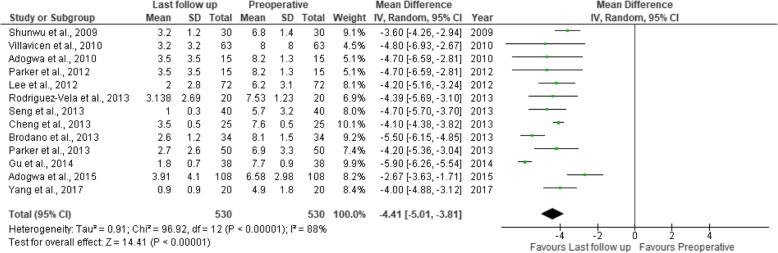
Forest plot of the study comparisons: comparison between preoperative and last follow-up visual analog scale scores for leg pain within the OTLIF group

**Fig. 15:**
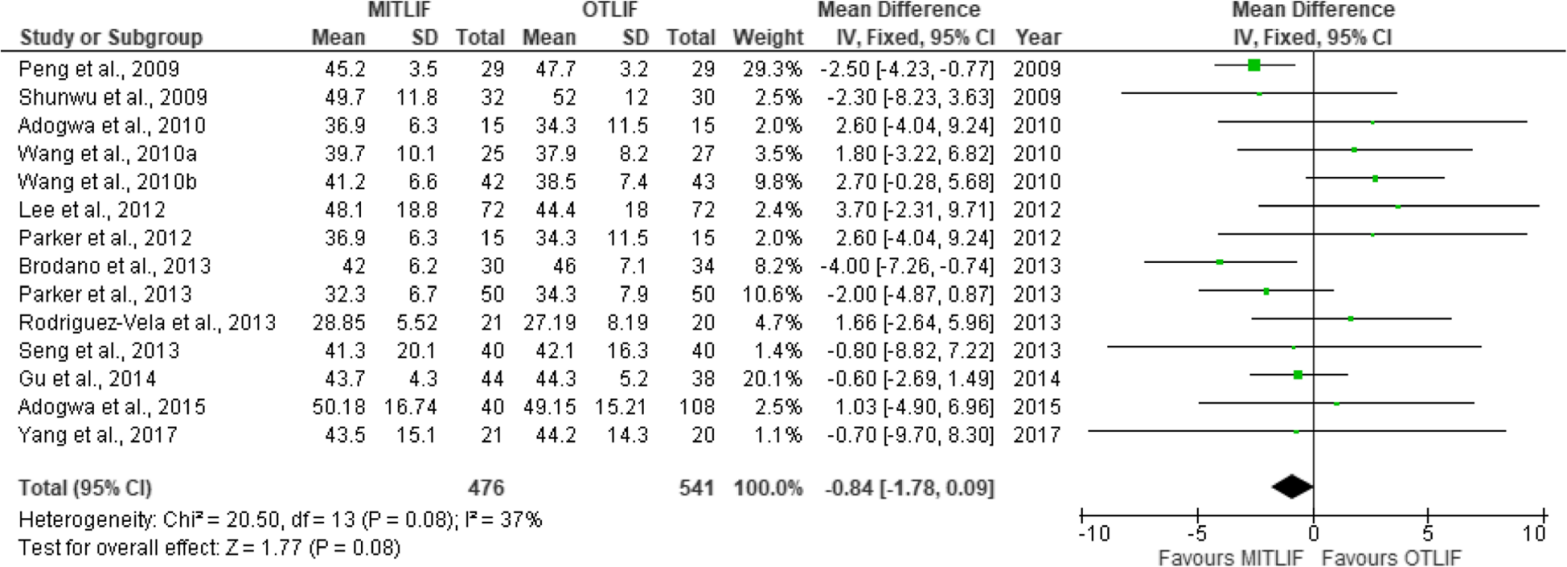
Forest plot of the study comparisons: comparison between MITLIF and OTLIF outcomes for preoperative Oswestry disability index (%)

**Fig. 16:**
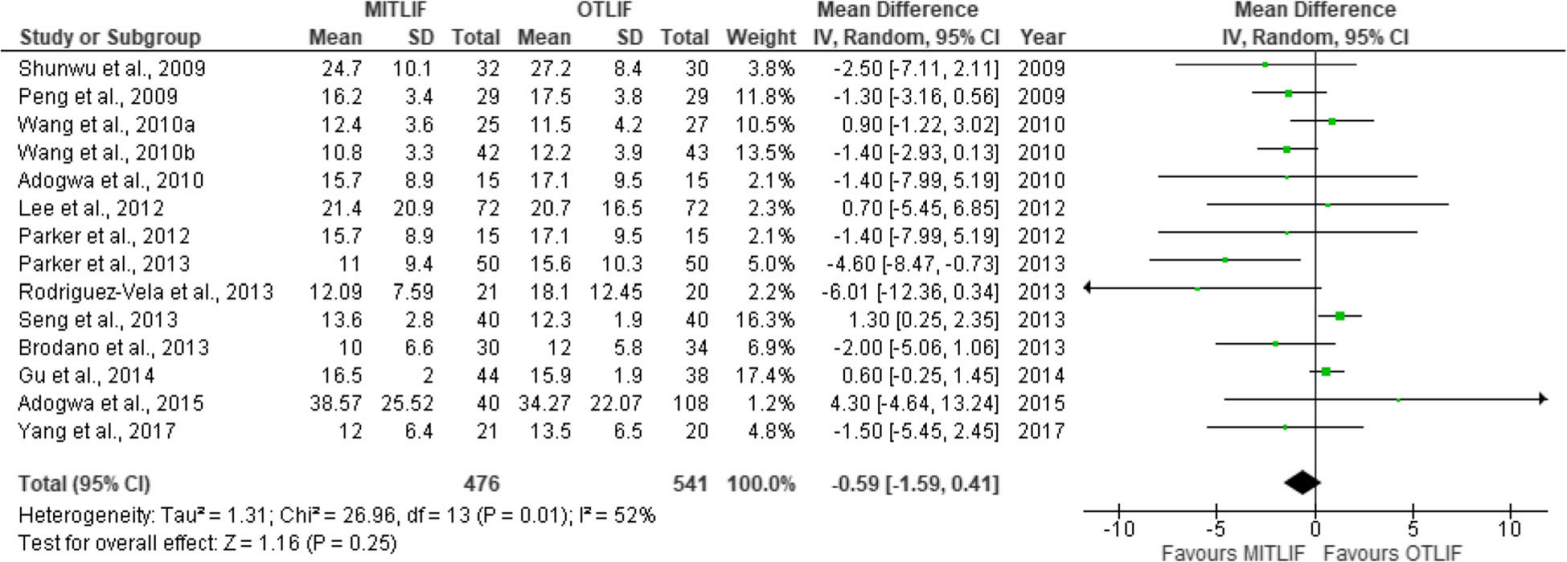
Forest plot of the study comparisons: comparison between MITLIF and OTLIF outcomes for Oswestry disability index (%) at last follow-up

**Fig. 17:**
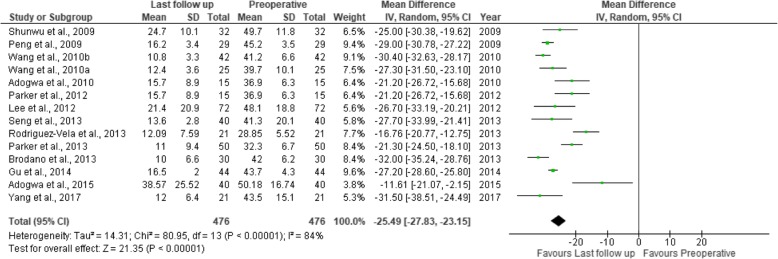
Forest plot of the study comparisons: comparison between preoperative and last follow-up Oswestry disability index (%) within the MITLIF group

**Fig. 18:**
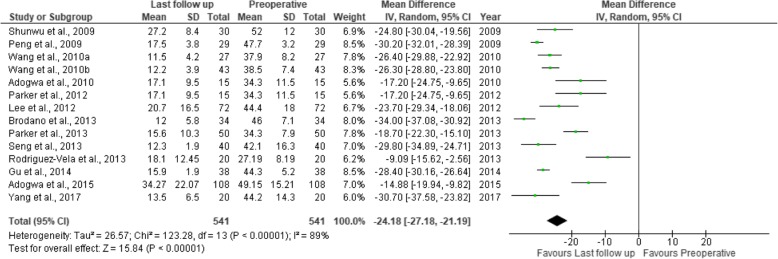
Forest plot of the study comparisons: comparison between preoperative and last follow-up Oswestry disability index (%) within the OTLIF group

Thirty-two studies were included in this review, including 18 prospective studies, 13 retrospective studies, and a single randomized controlled trial (Table 1). Our data are presented in Tables 2, 3, 4, 5, 6, 7, 8, and 9, and the results of the meta-analysis are shown in Figs. 1, 2, 3, 4, 5, 6, 7, 8, 9, 10, 11, 12, 13, 14, 15, 16, 17, and 18. The total number of patients was 2385, of which 1285 patients underwent MITLIF and 1100 patients underwent OTLIF. The total number of male patients was 1008 patients, of which 542 patients underwent MITLIF and 466 patients underwent OTLIF. The total number of female patients was 1299 patients, of which 704 patients underwent MITLIF and 595 patients underwent OTLIF. The mean age was 52.87 years in the MITLIF group and 54.19 years in the OTLIF group. The mean follow-up was 27.8 months.

**Table 1:** Studies

Author	Zhang et al. [14]	Yang et al. [15]	Tschugg et al. [16]	Kulkarni et al. [17]	Hey et al. [18]	Adogwa et al. [19]
Year of publication	2017	2017	2017	2016	2015	2015
Study design	Retrospective	RCT	Retrospective	Prospective	Prospective	Prospective
Level of evidence	–	II	–	–	–	–
Number of patients	107 (M = 48, O = 59)	41 (MI = 21, O = 20)	67 (MI = 19, O = 48)	61 (MI = 36, O = 25)	50 (MI = 25, O = 25)	148 (MI = 40, O = 108)
Follow-up (months)	–	Minimum 24 months	3 months	36.5 months (mean follow-up)	Minimum 24 months	24 months
Mean patient age (years)	MI: 55.7, O: 59.7	MI: 63.5, O: 58.0	MI: 63.9, O: 64.4	MI: 51.55, O: 50.4	MI: 43.6, O: 44.4	MI: 56.62, O: 56.12
Gender (m/f)	MI: 24/24, O: 32/27	MI: 7/14, O: 8/12	MI: 8/11, O: 16/32	MI: 10/26, O: 11/14	MI: 13/12, O: 13/12	MI: 20/20, O: 47/61
Diagnosis	-DDD (MI/O: 40/47) -Spinal instability (MI/O: 8/12)	-Spinal stenosis (MI/O: 11/9) -Olisthesis (MI/O: 5/6) -Disc herniation with segmental instability (MI/O: 5/5)	-Lumbar spondylodiscitis	-Olisthesis (MI/O: 30/12) -Disc herniation (MI/O: 5/11) -Lumbar canal stenosis (MI/O: 1/2)	-DDD: (MI/O: 2/2) -Prolapsed intervertebral discs (MI/O: 12/12) -Spinal stenosis (MI/O: 3/3) -Spondylolisthesis (MI/O: 7/7)	-DDD: (MI/O: 27/81) -Olisthesis (MI/O: 29/78)
Outcomes	Operative time (min), LOS (days), HBL, TBL, PBL	VAS (back, leg), ODI, operative time (min), interbody fusion (grade I—Bridwell criteria), HBL, TBL	Intraoperative blood transfusion, operative time, LOS, postoperative complications	VAS (back, leg), ODI, LOS, operative time, radiation exposure, QCRP, blood loss	Operation time, EBL, drop in hemoglobin on the first postoperative day, LOS, duration to ambulation, ODI, cage height and fusion rates, complications	VAS (back, leg), ODI, complications
Author	Yee et al. [20]	Terman et al. [21]	Wong et al. [22]	Sulaiman et al. [13]	Singh et al. [23]	Gu et al. [24]
Year of publication	2014	2014	2014	2014	2014	2014
Study design	Retrospective	Retrospective	Prospective	Retrospective	Prospective	Prospective
Level of evidence	–	–	–	–	–	–
Number of patients	68 (MI = 52, O = 16)	74 (MI = 53, O = 21)	198 (MI = 144, O = 54)	68 (MI = 57, O = 11)	66 (MI = 33, O = 33)	82 (MI = 44, O = 38)
Follow-up (months)	Minimum 6 months	30 months (mean follow-up)	45 months (mean follow-up)	–	–	20 months (mean follow-up)
Mean age (years)I	MI: 47.9, O: 56.1	MI: 52.4, O: 58.2	MI: 61, O: 58	MI: 61.1, O: 56.4	MI: 51.67, O: 49.85	MI: 66.4, O: 64.1
Gender (m/f)	MI: 24/28, O: 5/11	MI: 24/29, 0: 13/8	MI: 61/83, O: 25/29	MI: 17/40, O: 4/7	MI: 23/10, O: 21/12	MI: 19/25, O: 15/23
Diagnosis	-DDD (MI/O: 17/2) -Herniated disc (7/0) -Olisthesis (MI/O: 24/12) -Stenosis (MI/O: 4/2)	-DDD or spondylosis (MI/O: 10/5) -Herniated disc (MI/O: 3/0) -Olisthesis (MI/O: 32/14) -Stenosis (MI/O: 8/2)	-Olisthesis +/− tilt with stenosis -Post-laminectomy instability with stenosis -DDD	Grade 1–2 degenerative olisthesis	-DDD (MI/O: 19/19) -Olisthesis (MI/O: 6/9) -Spinal stenosis (MI/O: 8/5)	-DDD (MI/O: 15/11) -Two-level lumbar stenosis (MI/O: 18/14) -Lumbar stenosis with segmental instability (MI/O: 11/13)
Outcomes	Development of symptomatic ASD (defined by: 1. new back and/or leg pain, 2. imaging findings adjacent to original surgical level, 3. decision to treat)	VAS, ODI, EBL, LOS; in obese patients	Blood loss, operative time, VAS, ODI, LOS, inpatient institutional costs, radiation exposure, fusion rates, segmental lordosis correction, complications and revisions	Operation time, EBL, perioperative complications, LOS, VAS, ODI, hospital costs	Operation time, LOS, EBL, anesthesia time (minutes), VAS, hospital costs/ payment amount	Operation time, intraoperative blood loss, transfusion volume, LOS, radiation exposure time, VAS, ODI, fusion rates, complications
Author	Brodano et al. [25]	Seng et al. [26]	Cheng et al. [27]	Lau et al. [28]	Rodriguez-Vela et al. [29]	Parker et al. [30]
Year of publication	2013	2013	2013	2013	2013	2013
Study design	Retrospective	Retrospective	Retrospective	Retrospective	Prospective	Prospective
Level of evidence	–	III	–	–	–	–
Number of patients	64 (MI = 30, O = 34)	80 (MI = 40, O = 40)	75 (MI = 50, O = 25)	127 (MI = 78, O = 49)	41 (MI = 21, O = 20)	100 (MI = 50, O = 50)
Follow-up (months)	23 months (mean follow-up)	60 months	60 months (average follow-up)	–	Minimum 36 months	24 months
Mean age (years)	MI: 46, O: 51	MI: 56.6, O: 56.8	MI: 53.7, O:54.3	-Class I obesity (BMI 30.0–34.9 kg/m^2^): MI: 52.5, O:54.1 -Class II obesity (BMI 35.0–39.9 kg/m^2^): MI: 50.5, O: 57.4 -Class III obesity (BMI > 40): MI: 53.5, O: 59.4	MI: 41.81, O: 43.15	MI: 53.5, O: 52.6
Gender (m/f)	MI: 18/12, O: 20/14	MI: 7/33, O: 7/33	MI: 27/23, O: 14/11	MI: 38/40, O: 23/26	MI: 14/7, O: 13/7	MI: 16/34, O: 18/32
Diagnosis	-DDD -Grade I degenerative olisthesis	-Olisthesis (MI/O: 31/33) -DDD with spinal stenosis (MI/O: 9/7)	-Spondylosis (MI/O: 28/12) -Olisthesis (MI/O: 27/14) -> Grades I and II (MI/O: 27/12) -> Grades III and IV (MI/O: 0/2) -Foraminal stenosis (MI/O: 25/10)	-Olisthesis (MI/O: 50/25) -DDD alone (MI/O: 12/12) -DDD with stenosis (MI/O: 9/11) -DDD with deformity (MI/O: 1/0) -DDD with disc herniation (MI/O: 6/1)	-DDD	-Grade I degenerative olisthesis
Outcomes	VAS, ODI, TBL, LOS, operation time, complications	VAS, ODI, radiation exposure time, operative time, LOS, complication rate, fusion rates (Bridwell criteria)	Postoperative pain medication, functional ability, VAS, EBL, operative time, LOS, fusion rates, complications, inpatient hospitalization costs	EBL, complications (total, intraoperative and 30-day postoperative), LOS	VAS, NASS, ODI, SF-36 Health Survey, postoperative complications	VAS, ODI, SF-36 Health Survey, operative time, EBL, complications, LOS, narcotic independence, return to work, hospital costs (direct and indirect)
Author	Adogwa et al. [31]	Wang et al. [32]	Pelton et al. [33]	Lee et al. [6]	Parker et al. [34]	Lau et al. [35]
Year of publication	2012	2012	2012	2012	2012	2010
Study design	Prospective	Prospective	Prospective	Prospective	Prospective	Retrospective
Level of evidence	–	–	–	–	–	–
Number of patients	21 (MI = 14, O = 7)	81 (MI = 42, O = 39)	66 (MI = 33, O = 33)	144 (MI = 72, O = 72)	30 (MI = 15, O = 15)	22 (MI = 10, O = 12)
Follow-up (months)	24 months	36 months (mean follow-up)	–	24 months	24 months	Minimum 12 months
Mean age (years)	MI: 48.14, O: 47.28	MI: 56.4, O: 54.2	MI:51.67, O: 49.85	MI: 52.2, O: 56.6	MI: 50.8, O: 49.7	MI: 46.9, O: 56.9
Gender (m/f)	MI: 4/10, O: 3/4	MI: 13/29, O: 12/27	MI: 23/10, O: 21/12	MI: 20/52, O: 22/50	MI: 7/8, O: 5/10	MI: 4/6, O: 5/7
Diagnosis	-DDD -Grade I olisthesis	-Stenosis (MI/O: 23/20) -Olisthesis (MI/O: 14/15) -Postoperative instability (MI/O: 5/4)	-DDD (MI/O: 13/14) -Olisthesis (MI/O: 20/19)	-Olisthesis (grades I and II) -Recurrent prolapsed disc -Spinal stenosis -DDD	-Grade I degenerative olisthesis	-Spondylosis (MI/O: 5/6) -Olisthesis (MI/O: 4/6) -Spondylolysis (MI/O: 1/0)
Outcomes	VAS, ODI, SF-36 Health Survey, operative time, EBL, LOS, duration of narcotic use, time to return to work, CPK (preoperative; days 1and 7, and 1.5, 3, and 6 months postoperative)	Operative time, blood loss, X-ray exposure time, complications, fusion rates, VAS, ODI—in overweight and obese patients	Operative time, EBL, LOS, anesthesia time, VAS, direct and indirect costs—in WC and non-WC patients	VAS, ODI, SF-36 Health Survey, NASS, time to return to full function, operative time, EBL, radiation exposure time, complications, fusion rates (Bridwell grading system)	VAS, ODI, quality of life (EuroQuol-5D), duration of narcotic use, time to return to work, direct and indirect costs, operative time, EBL, complications, LOS, fusion rates	Operative time, blood loss, LOS, pain scores, blood transfusion, time to ambulation, complications
Author	Adogwa et al. [36]	Wang et al. [37]	Villavicencio et al. [14]	Shunwu et al. [38]	Wang et al. [37]	Peng et al. [39]
Year of publication	2010	2010	2010	2009	2010	2009
Study design	Retrospective	Prospective	Retrospective	Prospective	Prospective	Prospective
Level of evidence	–	–	–	–	–	–
Number of patients	30 (MI = 15, O = 15)	52 (MI = 25, O = 27)	139 (MI = 76, O = 63)	62 (MI = 32, O = 30)	85 (MI = 42, O = 43)	58 (MI = 29, O = 29)
Follow-up (months)	24 months	27.5 (mean follow-up)	37.5 (average follow-up)	24 months (minimum follow-up)	26.3 (mean follow-up)	24 months (minimum follow-up)
Mean age (years)	MI: 50.8, O: 49.7	MI: 54.8, O: 56.2	MI: 50.5, O: 58.9	MI: 51.4, O: 52.0	MI: 47.9, O: 53.2	MI: 54.1, O: 52.5
Gender (m/f)	MI: 7/8, O: 5/10	MI: 13/12, O: 15/12	MI: 45/31, O: 38/25	MI: 18/14, O: 14/16	MI: 13/29, O: 16/27	MI: 5/24, O: 5/24
Diagnosis	Grade I Olisthesis	-Recurrent disc herniation (MI/O: 7/8) -Lumbar canal stenosis(MI/O: 10/9) -Segmental instability (MI/O: 5/7) -Olisthesis < Grade II (MI/O: 3/3)	-DDD with/without disc herniation -Olisthesis -Stenosis at one or two spinal levels	-Discogenic low back pain (MI/O: 6/4) -Unilateral lumbar disc herniation (MI/O: 13/4) -Foraminal stenosis (MI/O: 3/8) -Separation of posterior ring apophysis (MI/O: 3/4) -Low-grade olisthesis (MI/O: 5/8) -Single-segment instability (MI/O: 2/2)	-Degenerative Olisthesis (MI/O: 24/22) -Isthmic Olisthesis (MI/O: 18/21)	-Grade I/II Olisthesis -DDD presenting with low back pain and radicular symptoms
Outcomes	VAS, ODI, EuroQol-5D, occupational disability, narcotic use, time to return to work, operative time, EBL	VAS, ODI, operative time, blood loss, radiation exposure time, complications, fusion rates	Operative time, EBL, LOS, VAS, patient satisfaction, MacNab’s criteria fusion rates, complications (major, minor)	Operative time, blood loss, total transfusion volume, LOS, time to ambulation, complications, serum CK, VAS, ODI, fusion rates	Operative time, transfusion volume, X-ray exposure times, LOS, complications, VAS, ODI, fusion rates	NASS, ODI, VAS, SF-36, operative time, blood loss, radiation exposure time, time to ambulation, narcotic use, fusion rates (Bridwell criteria)
Author	Dhall et al. [40]	Schizas et al. [41]				
Year of publication	2008	2008				
Study design	Retrospective	Prospective				
Level of evidence	–	–				
Number of patients	42 (MI = 21, O = 21)	36 (MI = 18, O = 18)				
Follow-up (months)	24 months (MI), 34 months (O) (mean follow-up)	22 months (MI), 24 months (O) (average follow-up)				
Mean age (years)	MI: 53, O: 53	MI: 45.5, O: 48.1				
Gender (m/f)	–	–				
Diagnosis	-DDD (MI/O: 14/10) -Degenerative olisthesis (MI/O: 7/11)	-Isthmic Olisthesis (MI/O: 15/6) -Asymmetrical disc disease with foraminal stenosis (MI/O: 2/12) -Iatrogenic spondylosis (MI/O: 1/0)				
Outcomes	Operative time, EBL, LOS, complications, fusion rates, mPS	Operative time, intraoperative and total blood loss, radiation exposure time, VAS and ODI scores, analgesia intake, fusion rates, complications, learning curve				

*TLIF* transforaminal lumbar interbody fusion, *MITLIF* minimally invasive transforaminal lumbar interbody fusion, *OTLIF* open transforaminal lumbar interbody fusion, *BMI* body mass index, *VAS* visual analogue scale, *ODI* Oswestry disability index, *TBL* total blood loss, *HBL* hidden blood loss, *PBL* postoperative blood loss, *LOS* length of hospital stay, *QCRP* quantitative C-reactive protein, *ASD* adjacent segment disease, *EBL* estimated blood loss, *NASS* North American Spine Society, *CPK* creatine phosphokinase, *WC* workers’ compensation, *CK* creatine kinase, *mPS* modified Prolo scale

**Table 2:** Operative time (min)

Author	MITLIF			OTLIF		
	Mean	SD	Nr. of Pat.	Mean	SD	Nr. of Pat.
Zhang et al. 2017 [14]	146	± 15	48	136	± 25	59
Yang et al. 2017 [15]	179.0	± 20.7	21	141.8	± 18.8	20
Tschugg et al. 2017 [16]	173.4	± 71	19	208.8	± 86	48
Kulkarni et al. 2016 [17]	204	± 32.4	36	177.6	± 34.2	25
Hey et al. 2015 [18]	366.3	–	25	252.5	–	25
Wong et al. 2014 [22]	123	–	144	225	–	54
Sulaiman et al. 2014 [13]	375	± 14	57	161	± 7.6	11
Singh et al. 2014 [23]	115.8	± 28.2	33	186.0	± 31.0	33
Gu et al. 2014 [24]	195.5	± 28.0	44	186.6	± 23.4	38
Brodano et al. 2013 [25]	144	–	30	102	–	34
Seng et al. 2013 [26]	185	± 8.7	40	166	± 7	40
Cheng et al. 2013 [27]	244.6	± 73.0	50	278.8	± 14.5	25
Parker et al. 2013 [30]	274	–	50	229	–	50
Adogwa et al. 2012 [31]	235	± 88.36	14	211	± 43.23	7
Wang et al. 2012 [32]	127	± 25	42	168	± 37	39
Pelton et al. 2012 [33]	113	± 32.30	33	184.5	± 33.94	33
Lee et al. 2012 [6]	166.4	± 52.1	72	181.8	± 45.4	72
Parker et al. 2012 [34]	300	–	15	210	–	15
Lau et al., 2010 [35]	389.67	–	10	365.30	–	12
Adogwa et al. 2010 [36]	300	–	15	210	–	15
Wang et al. 2010 [37]	139	± 27	25	143	± 35	27
Villavicen et al. 2010 [42]	222.5	± 67.5	76	214.9	± 60	63
Shunwu et al. 2009 [38]	159.2	± 21.7	32	142.8	± 22.5	30
Wang et al. 2010 [37]	156	± 32	42	145	± 27	43
Peng et al. 2009 [39]	216.4	–	29	170.5	–	29
Dhall et al. 2008 [26]	199	–	21	237	–	21
Schizas et al. 2008 [41]	348	–	18	312	–	18

*MITLIF* minimally invasive transforaminal lumbar interbody fusion, *OTLIF* open transforaminal lumbar interbody fusion, *SD* standard deviation, *Nr* number, *Pat* patients

**Table 3:** Blood loss (ml)

Author	MITLIF			OTLIF		
	Mean	SD	Nr. of Pat.	Mean	SD	Nr. of Pat.
Zhang et al. 2017 [14]	602	± 251	48	742	± 275	59
Yang et al. 2017 [15]	355.3	± 75.0	21	538.6	± 129.5	20
Tschugg et al. 2017 [16]	110.5	± 205	19	472.3	± 555	48
Kulkarni et al. 2016 [17]	111.81	–	36	358.8	–	25
Hey et al., 2015 [18]	362.5	–	25	267.5	–	25
Terman et al. 2014 [21]	100	–	53	450	–	21
Wong et al. 2014 [22]	115	–	144	485	–	54
Sulaiman et al. 2014 [13]	95	± 20	57	786	± 107	11
Singh et al. 2014 [23]	124.4	± 92.0	33	380.3	± 191.2	33
Gu et al. 2014 [24]	248.4	± 94.3	44	576.3	± 176.2	38
Brodano et al. 2013 [25]	230	–	30	620	–	34
Seng et al. 2013 [26]	127.3	± 45.7	40	405	± 80	40
Cheng et al. 2013 [27]	392.5	± 284.0	50	535.5	± 324.0	25
Lau et al. 2013 [28]	168.6	± 162.1	78	661.0	± 561.3	49
Parker et al. 2013 [30]	200	–	50	350	–	50
Adogwa et al. 2012 [31]	220	± 207.32	14	280	± 219.65	7
Wang et al. 2012 [32]	326	± 122	42	835	± 247	39
Pelton et al. 2012 [33]	125.5	± 82.425	33	271	± 84.915	33
Lee et al. 2012 [6]	50.6	± 161.0	72	976.3	± 760.8	72
Parker et al. 2012 [34]	200	–	15	295	–	15
Lau et al. 2010 [35]	466.67	–	10	565,63	–	12
Adogwa et al. 2010 [36]	200	–	15	295	–	15
Wang et al. 2010 [37]	316	± 96	25	799	± 208	27
Villavicen et al. 2010 [42]	163.0	± 131.2	76	366.8	± 298.2	63
Shunwu et al. 2009 [38]	578	± 138.8	32	711.4	± 157.3	30
Wang et al. 2010 [37]	303	± 101	42	831	± 210	43
Peng et al. 2009 [39]	150	–	29	681	–	29
Dhall et al. 2008 [40]	194	–	21	505	–	21
Schizas et al. 2008 [41]	551	–	18	1438	–	18

*MITLIF* minimally invasive transforaminal lumbar interbody fusion, *OTLIF* open transforaminal lumbar interbody fusion, *SD* standard deviation, *Nr*. number, *Pat*. patients

**Table 4:** Length of hospital stay (days)

Author	MITLIF			OTLIF		
	Mean	SD	Nr. of Pat.	Mean	SD	Nr. of Pat.
Zhang et al. 2017 [14]	7.9	± 2.8	48	10.1	± 3.2	59
Tschugg et al. 2017 [16]	13.7	± 5	19	19.1	± 12	48
Kulkarni et al. 2016 [17]	4.11	± 1.8	36	5.84	± 2.249	25
Hey et al. 2015 [18]	10.0	–	25	7.7	–	25
Terman et al. 2014 [21]	2	–	53	3	–	21
Wong et al. 2014 [22]	2.75	–	144	4.40	–	54
Sulaiman et al. 2014 [13]	3.6	± 1	57	3.2	± 0.2	11
Singh et al. 2014 [23]	2.3	± 1.2	33	2.9	± 1.1	33
Gu et al. 2014 [24]	9.3	± 3.7	44	12.1	± 3.6	38
Brodano et al. 2013 [25]	4.1	–	30	7.4	–	34
Seng et al. 2013 [26]	3.6	± 0.3	40	5.9	± 0.4	40
Cheng et al. 2013 [27]	4.8	± 1.8	50	6.05	± 1.8	25
Lau et al. 2013 [28]	3.1	± 1.7	78	4.7	± 2.1	49
Parker et al. 2013 [30]	3	–	50	4	–	50
Adogwa et al. 2012 [31]	3	–	14	4	–	7
Pelton et al. 2012 [33]	2	± 0.713	33	3	± 1.1	33
Lee et al. 2012 [6]	3.2	± 2.9	72	6.8	± 3.4	72
Parker et al. 2012 [34]	3.0	–	15	5.0	–	15
Lau et al. 2010 [35]	5.00	–	10	6.17	–	12
Villavicen et al. 2010 [42]	3.0	± 2.3	76	4.2	± 3.5	63
Shunwu et al. 2009 [38]	9.3	± 2.6	32	12.50	± 1.8	30
Wang et al. 2010 [37]	10.6	± 2.5	42	14.6	± 3.8	43
Peng et al. 2009 [39]	4.0	–	29	6.7	–	29
Dhall et al. 2008 [40]	3	–	21	5.5	–	21
Schizas et al. 2008 [41]	6.1	–	18	8.2	–	18

*MITLIF* minimally invasive transforaminal lumbar interbody fusion, *OTLIF* open transforaminal lumbar interbody fusion, *SD* standard deviation, *Nr.* number, *Pat.* patients

**Table 5:** Complication rate

Author	MITLIF		OTLIF	
	Number of complications	Number of patients	Number of complications	Number of patients
c et al. 2017 [15]	2	21	1	20
Hey et al. 2015 [18]	8	25	2	25
Adogwa et al. 2015 [19]	5	40	12	108
Terman et al. 2014 [21]	9	53	11	21
Sulaiman et al. 2014 [13]	4	57	2	11
Gu et al. 2014 [24]	5	44	4	38
Brodano et al. 2013 [25]	1	30	2	34
Seng et al. 2013 [26]	2	40	4	40
Lau et al. 2013 [28]	9	78	14	49
Adogwa et al. 2012 [31]	0	14	2	7
Wang et al. 2012 [32]	4	42	7	39
Parker et al. 2012 [34]	0	15	0	15
Lau et al. 2010 [35]	4	10	1	12
Adogwa et al. 2010 [36]	0	15	0	15
Shunwu et al. 2009 [38]	6	32	5	30
Peng et al. 2009 [39]	2	29	4	29
Dhall et al. 2008 [40]	3	21	2	21

*MITLIF* minimally invasive transforaminal lumbar interbody fusion, *OTLIF* open transforaminal lumbar interbody fusion

**Table 6:** Radiation exposure time (s)

Author	MITLIF			OTLIF		
	Mean	SD	Number of patients	Mean	SD	Number of patients
Gu et al. 2014 [24]	45.3	± 11.7	44	28.9	± 8.2	38
Seng et al. 2013 [26]	55.2	± 11.3	40	16.4	± 2.1	40
Wang et al. 2012	46	± 21	42	24	± 8	39
Lee et al. 2012 [6]	49.0	± 33.9	72	17.6	± 20.0	72
Wang et al. 2010 [37]	73	± 21	25	39	± 16	27
Wang et al. 2010 [37]	84	± 21	42	37	± 19	43
Peng et al. 2009 [39]	105.5	–	29	35.2	–	29

*MITLIF* minimally invasive transforaminal lumbar interbody fusion, *OTLIF* open transforaminal lumbar interbody fusion, *SD* standard deviation

**Table 7:** Visual analog scale scores for back pain

Author	MITLIF			OTLIF		
	Nr. of Pat.	Preoperative (mean ± SD)	Last follow-up (mean ± SD)	Nr. of Pat.	Preoperative (mean ± SD)	Last follow-up (mean ± SD)
Yang et al. 2017 [15]	21	5.8 ± 0.9	1.0 ± 0.9	20	5.6 ± 0.8	1.2 ± 1.2
Adogwa et al. 2015 [19]	40	6.97 ± 2.49	4.55 ± 3.81	108	7.0 ± 2.44	4.67 ± 3.67
Terman et al. 2014 [21]	53	7.1 (−)	4.7 (−)	21	7.1 (−)	4.3 (−)
Wong et al. 2014 [22]	144	6.37 (−)	1.05 (−)	54	6.72 (−)	1.70 (−)
Sulaiman et al. 2014 [13]	57	7.3 (−)	3.2 (−)	11	7.3 (−)	5.1 (−)
Gu et al. 2014 [24]	44	7.3 ± 1.2	1.9 ± 0.7	38	7.4 ± 1.0	1.8 ± 0.6
Brodano et al. 2013 [25]	30	7.8 ± 1.4	2.3 ± 1.3	34	8.1 ± 1.5	2.6 ± 1.2
Seng et al. 2013 [26]	40	5.6 ± 3.3	1.3 ± 0.4	40	6.2 ± 2.7	0.9 ± 0.3
Cheng et al. 2013 [27]	50	7.1 ± 0.7	2.9 ± 0.3	25	7.6 ± 0.5	3.5 ± 0.5
Rodriguez-Vela et al. 2013 [29]	21	7.04 ± 1.12	3.381 ± 2.69	20	7.19 ± 2.21	4.611 ± 3.12
Parker et al. 2013 [30]	50	8.1 ± 2.6	3.3 ± 2.9	50	8.5 ± 2.2	3.6 ± 2.8
Adogwa et al. 2012 [31]	14	6.80 ± 2.40	–	7	6.14 ± 1.67	3.14 (−)
Wang et al. 2012 [32]	42	6.3 ± 2.5	1.3 ± 0.6	39	6.0 ± 2.1	1.5 ± 0.5
Lee et al. 2012 [6]	72	6.3 ± 2.9	2.3 ± 3.0	72	6.3 ± 2.9	2.4 ± 2.7
Parker et al. 2012 [34]	15	8.4 ± 1.7	5.5 ± 2.6	15	9.3 ± 0.9	4.7 ± 3.2
Adogwa et al. 2010 [36]	15	8.4 ± 1.7	5.5 ± 2.6	15	9.3 ± 0.9	4.7 ± 3.2
Wang et al. 2010 [37]	25	7.1 ± 2.4	1.3 ± 0.5	27	6.9 ± 1.7	1.5 ± 0.4
Villavicen et al. 2010 [42]	76	7.4 (−)	3.4 (−)	63	8.0 (−)	3.2 (−)
Shunwu et al. 2009 [38]	32	6.8 ± 1.2	2.3 ± 1.5	30	6.8 ± 1.4	3.2 ± 1.2
Wang et al. 2010 [37]	42	7.2 ± 2.1	0.92 ± 0.5	43	7.4 ± 1.6	1.1 ± 0.6
Peng et al. 2009 [39]	29	6 (−)	1 (−)	29	6.5 (−)	1.2 (−)
Schizas et al. 2008 [41]	18	7.7 (−)	3.5 (−)	18	5.0 (−)	2.8 (−)

*MITLIF* minimally invasive transforaminal lumbar interbody fusion, *OTLIF* Open transforaminal lumbar interbody fusion, *Nr.* number, *Pat.* patients, *SD* standard deviation

**Table 8:** Visual analog scale scores for leg pain

Author	MITLIF			OTLIF		
	Nr. of Pat.	Preoperative (mean ± SD)	Last follow-up (mean ± SD)	Nr. of Pat.	Preoperative (mean ± SD)	Last follow-up (mean ± SD)
Yang et al. 2017 [15]	21	5.2 ± 1.3	0.6 ± 0.7	20	4.9 ± 1.8	0.9 ± 0.9
Adogwa et al. 2015 [19]	40	7.07 ± 3.00	3.3 ± 4.53	108	6.58 ± 2.98	3.91 ± 4.10
Terman et al. 2014 [21]	53	7.1 (−)	4.7 (−)	21	7.1 (−)	4.3 (−)
Wong et al. 2014 [22]	144	8.9 (−)	1.15 (−)	54	8.82 (−)	1.30 (−)
Sulaiman et al. 2014 [13]	57	7.3 (−)	3.2 (−)	11	7.3 (−)	5.1 (−)
Gu et al. 2014 [24]	44	7.6 ± 0.9	1.7 ± 0.6	38	7.7 ± 0.9	1.8 ± 0.7
Brodano et al. 2013 [25]	30	7.8 ± 1.4	2.3 ± 1.3	34	8.1 ± 1.5	2.6 ± 1.2
Seng et al. 2013 [26]	40	5.9 ± 2.8	0.8 ± 0.4	40	5.7 ± 3.2	1.0 ± 0.3
Cheng et al. 2013 [27]	50	7.1 ± 0.7	2.9 ± 0.3	25	7.6 ± 0.5	3.5 ± 0.5
Rodriguez-Vela et al. 2013 [29]	21	7.31 ± 2.05	2.381 ± 2.65	20	7.53 ± 1.23	3.138 ± 2.69
Parker et al. 2013 [30]	50	6.5 ± 3.6	3.0 ± 3.0	50	6.9 ± 3.3	2.7 ± 2.6
Adogwa et al. 2012 [31]	14	5.99 ± 2.61	–	7	6.07 ± 2.69	1.58 (−)
Lee et al. 2012 [6]	72	5.8 ± 3.3	1.6 ± 2.7	72	6.2 ± 3.1	2.0 ± 2.8
Parker et al. 2012 [34]	15	8.5 ± 1.3	5.5 ± 2.9	15	8.2 ± 1.3	3.5 ± 3.5
Adogwa et al. 2010 [36]	15	8.5 ± 1.3	5.5 ± 2.9	15	8.2 ± 1.3	3.5 ± 3.5
Wang et al. 2010 [37]	25	–	1.0 ± 0.3	27	–	1.3 ± 0.4
Villavicen et al. 2010 [42]	76	7.4 (−)	3.4 (−)	63	8.0 (−)	3.2 (−)
Shunwu et al. 2009 [38]	32	6.8 ± 1.2	2.3 ± 1.5	30	6.8 ± 1.4	3.2 ± 1.2
Peng et al. 2009 [39]	29	7 (−)	1 (−)	29	6.5 (−)	1.1 (−)
Schizas et al. 2008 [41]	18	7.7 (−)	3.5 (−)	18	5.0 (−)	2.8 (−)

*MITLIF* minimally invasive transforaminal lumbar interbody fusion, *OTLIF* open transforaminal lumbar interbody fusion, *Nr.* number, *Pat.* patients, *SD* standard deviation

**Table 9:** Oswestry Disability Index (%)

Author	MITLIF			OTLIF		
	Nr. of Pat.	Preoperative (mean ± SD)	Last follow-up (mean ± SD)	Nr. of Pat.	Preoperative (mean ± SD)	Last follow-up (mean ± SD)
Yang et al. 2017 [15]	21	43.5 ± 15.1	12.0 ± 6.4	20	44.2 ± 14.3	13.5 ± 6.5
Adogwa et al. 2015 [19]	40	50.18 ± 16.74	38.57 ± 25.52	108	49.15 ± 15.21	34.27 ± 22.07
Terman et al. 2014 [21]	53	59 (−)	44 (−)	21	58 (−)	45 (−)
Wong et al. 2014 [22]	144	52.8 (−)	18 (−)	54	51.2 (−)	21 (−)
Sulaiman et al. 2014 [13]	57	53.7 (−)	26.4 (−)	11	57.8 (−)	46.1 (−)
Gu et al. 2014 [24]	44	43.7 ± 4.3	16.5 ± 2.0	38	44.3 ± 5.2	15.9 ± 1.9
Brodano et al. 2013 [25]	30	42 ± 6.2	10 ± 6.6	34	46 ± 7.1	12 ± 5.8
Seng et al. 2013 [26]	40	41.3 ± 20.1	13.6 ± 2.8	40	42.1 ± 16.3	12.3 ± 1.9
Rodriguez-Vela et al. 2013 [29]	21	28.85 ± 5.52	12.09 ± 7.59	20	27.19 ± 8.19	18.10 ± 12.45
Parker et al. 2013 [30]	50	32.3 ± 6.7	11.0 ± 9.4	50	34.3 ± 7.9	15.6 ± 10.3
Adogwa et al. 2012 [31]	14	20.50 ± 7.76	–	7	22.57 ± 9.32	11.93 (−)
Wang et al. 2012 [32]	42	41.1 ± 10.3	18.2 ± 5.9	39	40.2	17.4 ± 7.1
Lee et al. 2012 [6]	72	48.1 ± 18.8	21.4 ± 20.9	72	44.4 ± 18.0	20.7 ± 16.5
Parker et al. 2012 [34]	15	36.9 ± 6.3	15.7 ± 8.9	15	34.3 ± 11.5	17.1 ± 9.5
Adogwa et al. 2010 [36]	15	36.9 ± 6.3	15.7 ± 8.9	15	34.3 ± 11.5	17.1 ± 9.5
Wang et al. 2010 [37]	25	39.7 ± 10.1	12.4 ± 3.6	27	37.9 ± 8.2	11.5 ± 4.2
Shunwu et al. 2009 [38]	32	49.7 ± 11.8	24.7 ± 10.1	30	52 ± 12	27.2 ± 8.4
Wang et al. 2010 [37]	42	41.2 ± 6.6	10.8 ± 3.3	43	38.5 ± 7.4	12.2 ± 3.9
Peng et al. 2009 [39]	29	45.2 ± 3.5	16.2 ± 3.4	29	47.7 ± 3.2	17.5 ± 3.8
Schizas et al. 2008 [41]	18	55 (−)	33 (−)	18	53 (−)	26 (−)

*MITLIF* minimally invasive transforaminal lumbar interbody fusion, *OTLIF* Open transforaminal lumbar interbody fusion, *Nr.* number, *Pat.* patients, *SD* standard deviation

### Operative time

Twenty-seven studies had sufficient data regarding the operative time (Table 2). The mean operative time was 214.69 min in the MITLIF group vs. 198.03 min in the OTLIF group. Based on our meta-analysis, the difference was not significant (*P* = 0.78) (Fig. 2).

### Blood loss

Twenty-nine studies had sufficient data regarding the amount of blood loss (Table 3). The mean blood loss volume was 247.82 ml in the MITLIF group vs. 568.18 ml in the OTLIF group. The difference was significant (*P* < 0.00001) (Fig. 3).

### Length of hospital stay

Twenty-five studies had sufficient information on length of hospital stay (LOS) (Table 4). The mean LOS was 5.05 days in the MITLIF group vs. 6.92 days in the OTLIF group. The difference was significant (*P* < 0.00001) (Fig. 4).

### Complications

The number of complications was identified in 17 studies (Table 5). The complication rate was 11.3% in the MITLIF group vs. 14.2% in the OTLIF group. The difference was not statistically significant (*P* = 0.05) (Fig. 5).

### Radiation exposure time

Data regarding radiation exposure time (Table 6) was identified in only seven studies. The mean radiation exposure time was 65.4 s in the MITLIF group vs. 28.3 s in the OTLIF group. The difference was significant (*P* < 0.00001) (Fig. 6).

### Visual analogue scale score (back and leg)

Twenty-two studies had sufficient data regarding the visual analogue scale (VAS) scores. The mean preoperative VAS score for back pain was 7.04 in the MITLIF group vs. 7.10 in the OTLIF group, with a statistically significant difference (*P* = 0.002). The mean VAS score for back pain at the final follow-up was 2.69 in the MITLIF group vs. 2.88 in the OTLIF group; the difference was not significant (*P* = 0.15). The mean preoperative VAS score for leg pain was 7.13 in the MITLIF group vs. 7.01 in the OTLIF group, with a statistically significant difference (*P* = 0.0.1). The mean VAS score for leg pain at the final follow-up was 2.62 in both groups (Tables 7 and 8, Figs. 7, 8, 9, 10, 11, 12, 13, and 14).

### Oswestry Disability Index

Twenty studies contained sufficient data on the Oswestry Disability Index (ODI) scores, expressed in percent. The mean preoperative ODI score was 43.08 in the MITLIF group vs. 42.95 in the OTLIF group; the difference was not significant. The mean ODI score at the final follow-up was 19.48 in the MITLIF group vs. 20.62 in the OTLIF group, and the difference was not significant (*P* = 0.25) (Table 9, Figs. 15, 16, 17, and 18).

## Discussion

Since the introduction of MITLIF in the early 2000s by Foley et al. as an alternative to traditional OTLIF, several studies have compared both techniques for perioperative, postoperative, clinical, and radiological outcomes. The parameters that have been compared most often between the two techniques are operative time, blood loss, LOS, complication rate, radiation exposure time, and various pain scores. Other items include fusion rates, clinical and radiological outcomes in selected groups of patients, e.g., overweight patients, and the costs involved in both procedures to evaluate the cost-effectiveness of the techniques. Our literature review identified obvious trends for evaluating certain parameters, such as blood loss, LOS, and radiation exposure time. On the other hand, other parameters, such as operative time and complication rate, remain highly controversial when comparing MITLIF and OTLIF.

Among our included studies, 28 studies compared blood loss between patients undergoing MITLIF and OTLIF, of which 26 studies showed that blood loss was significantly lower in the MITLIF group. According to Lau et al. [28], this applies also to obese patients. The authors conducted a retrospective study in 2013 where 127 obese patients (body mass index (BMI) of at least 30 kg/m^2^) who underwent single-level TLIF were retrospectively identified. Results showed that MITLIF was associated with significantly less blood loss in the three identified obesity classes [28]. However, another study by Lau and colleagues in 2010 showed that perioperative blood loss volume was similar between MITLIF and OTLIF, although more patients undergoing OTLIF required perioperative transfusions [44]. In contrast, Hey and colleagues examined 50 patients and showed no significant difference in blood loss for single-level TLIF between MITLIF and OTLIF, but found higher blood loss volumes in two-level MITLF compared with two-level OTLIF [19].

LOS was discussed in 25 of the studies included in our review. Twenty-three of these studies showed that LOS was significantly shorter in the MITLIF group. However, Sulaiman et al. [13] and Lau et al. [44] showed no significant difference in LOS between MITLIF and OTLIF patients. Hey and Hee showed no significant difference in LOS for single-level TLIF when comparing MITLIF and OTLIF, but found longer LOS for two-level MITLIF vs. two-level OTLIF [18]. Although the results of studying these parameters favor MITLIF, the prolonged radiation exposure time involved in MITLIF is considered a drawback. Ten of our studies discussed radiation exposure time, of which nine studies showed significantly higher radiation exposure time for MITLIF [6, 17, 22, 24, 26, 39, 45, 46]. Gu et al. [24] suggested that the smaller operative field, lack of visualization of the bony landmarks, and the steep learning curve associated with MITLIF explains the prolonged radiation exposure time. Based on our experience, the prolonged fluoroscopy time needed for placing pedicle screws percutaneously with MITLIF is the primary contributor to the higher radiation exposure compared with OTLIF. In our institution, when performing OTLIF, the pedicle screws are placed free-hand, and minimal radiation is needed because screw position is confirmed with fluoroscopy usually only once, after all screws have been placed. On the other hand, in MITLIF, radiation can be needed more than once, when placing each pedicle screw, to ensure correct screw positioning. The cumulative effects of radiation exposure on the patient and the operating team should not be ignored [45]. Seng et al. [26] and Wang et al. [45, 46] suggested that with greater surgical experience, radiation exposure time could be reduced. A prospective study conducted by Schizas et al. to evaluate their initial experience comparing perioperative outcomes between 18 MITLIF patients and 18 OTLIF patients showed no significant difference in radiation exposure time between the two groups [2].

Operative time is another area of interest when comparing MITLIF and OTLIF techniques, which was discussed in most studies comparing the two procedures. Twenty-seven studies from the included studies in our review compared operative time between MITLIF and OTLIF; 13 studies showed longer operative time for MITLIF. Kulkarni et al. [17] conducted a prospective study examining 61 patients and showed longer operative time for MITLIF, which was considered secondary to the steep learning curve associated with MITLIF. Hey and Hee in their prospective study examining 50 patients, also showed significantly longer operative time for MITLIF [18]. This was explained by the technically more demanding MITLIF as a result of the limited visibility of the surgical field. These findings concur with those of Peng et al. [39], where the more technically challenging MITLIF explained the significantly longer operative time. Other authors showed no significant difference in operative time between MITLIF and TLIF [16, 17, 26, 37, 40, 42, 46, 47]. On the other hand, six of our included studies showed shorter operative time for MITLIF [6, 22, 23, 27, 33, 45]. Wong et al. reported in their prospective study examining 198 patients, of which 144 patients underwent MITLIF, significantly shorter operative time for MITLIF [22]. This was because the 144 MITLIF procedures (2006–2008) were well past the initial learning curve of the first 100 MITLIF procedures performed from 2002 to 2004. These results are similar to the findings of Brodano et al. [25] who retrospectively examined 64 patients, of which 30 patients underwent MITLIF. Although the surgical time was longer for MITLIF overall, there was a statistically significant difference in operative time between the initial 15 MITLIF patients (mean time, 3.2 h) and the latter 15 patients (mean time, 1.8 h), which confirms that MITLIF requires a learning curve, and once adequate experience is gained, operative time decreases significantly [25].

The complication rate with MITLIF is controversial when comparing MITLIF and OTLIF with heterogeneity seen among our included studies. Many studies showed no significant difference in the incidence of complications between the techniques [16, 19, 24, 26, 31, 48, 49]. Adogwa et al. [19] conducted a prospective study examining 148 patients who underwent either MITLIF or OTLIF. Complications associated with both techniques included wound infection, nerve root injury, and durotomy. Hardware failure was also reported with both techniques; however, no statistically significant difference between techniques was reported in one study [19]. Other studies reported significantly higher complication rates with OTLIF [13, 21, 22, 28, 39]. Terman et al. [26] retrospectively examined 74 obese patients (BMI > 30 kg/m^2^) and showed significantly higher complication rates with OTLIF. Complications included general cardiopulmonary complications, durotomy, and wound infection [21]. Wong et al. [22] prospectively studied 198 patients and found a significantly lower rate of systemic respiratory and urinary infections, which was attributed to patients’ overall earlier mobilization and ambulation, and a significantly lower overall wound infection rate with MITLIF. These lower rates were attributed to less tissue trauma, lower blood loss volumes, less need for drainage, and a smaller potential dead space [22]. Lau et al. retrospectively evaluated 127 obese patients (BMI of at least 30 kg/m^2^), and showed a significantly higher complication rate with OTLIF where patients undergoing OTLIF experienced 36% more complications [28]. In contrast, other studies showed higher complications in patients undergoing MITLIF [44, 18, 40, 42]. Lau et al. [27] showed that the complication rate tended to be higher for MITLIF, explained by higher technical demands with MITLIF, invisibility of the standard landmarks, and the steep learning curve. Dhall et al. retrospectively examined 42 patients and reported a higher rate of complication with MITLIF, including screw misplacement and cage migration, and attributed the higher rates to the steep learning curve and the higher technical demands [40].

Regarding clinical outcomes, measured using the VAS and ODI scores, most of our included studies showed significant improvement in VAS (for back and leg pain) and ODI scores for both MITLIF and OTLIF, with no significant difference between techniques at the final follow-up [6, 13, 15, 17–19, 21, 24–27, 29, 31, 33, 37, 39, 40, 42, 44–48, 50]. Considering direct postoperative pain, patients who underwent MITLIF experienced significantly less postoperative pain [16, 24–26, 37, 39, 45] with significantly less use of narcotic medications [39, 47, 48, 50]. This suggests improved early clinical outcomes in favor of MITLIF and explains the early ambulation, early return to work, and reduced LOS with MITLIF. However, studies reported no significant difference in the long-term clinical outcomes between the two techniques.

All included studies showed no statistically significant difference in fusion rates between MITLIF and OTLIF [6, 15, 18, 26, 37, 39, 42, 46, 47]. Villavicencio et al.’s retrospective study examining 139 patients reported successful fusion in all patients in both groups with no difference between MITLIF and OTLIF [42]. Seng et al. retrospectively examined 80 patients with 40 patients undergoing MITLIF and 40 patients undergoing OTLIF [26]. Patients undergoing OTLIF showed slightly better fusion at 6 months and 2 years compared with patients undergoing MITLIF but with no statistically significant difference. However, similar fusion rates in the two groups were achieved at the 5-year follow-up [26].

We also considered obese patients, which are a challenging group of patients when undergoing lumbar spine surgery [19, 28, 45]. Adogwa et al. [19] showed no significant difference in patient-reported outcomes for back pain, leg pain, and functional status 1 and 2 years postoperatively, or in the incidence of postoperative complications between morbidly obese patients undergoing MITLIF or OTLIF. These findings are similar to the results of Terman et al. [21], who showed significant improvement in pain and function in obese patients undergoing MITLIF and OTLIF, with comparable results to non-obese patients; also with no significant difference between MITLIF and OTLIF. Wang et al. [45] also showed promising results in favor of MITLF with significantly lower operative time and blood loss, significantly less postoperative pain, and significantly improved VAS and ODI scores 2 years postoperatively with no significant difference compared with obese patients in patients undergoing OTLIF. These findings indicate that MITLIF is a safe alternative for lumbar fusion in obese patients and provides similar clinical outcomes to OTLIF with no difference in postoperative complication rates [19] but also offers the benefits of less iatrogenic tissue injury and lower blood loss volumes and operative time [45].

A long-term risk after lumbar fusion is adjacent segment disease. Yee et al. [20] retrospectively examined 68 patients, of which 52 patients underwent MITLIF, and showed that the risk of ASD did not differ significantly between MITLIF and OTLIF. However, the MITLIF group showed a nonsignificant trend toward a decreased risk of ASD compared with the OTLIF group, suggesting that MITLIF may be associated with lower long-term morbidity compared with OTLIF [20]. These findings concur with the results of Wong et al., who showed decreased adjacent-level reoperation for MITLIF at 4 years [22].

Spondylodiscitis, a disease with increasing incidence, is a life-threatening condition with high mortality rates [51] for which spinal fusion is indicated in certain conditions including neurological deficits, instability, deformity, medically intractable pain, or disease progression [52, 53]. Patients suffering from spondylodiscitis, primarily patients with severe morbidity, greatly benefit from a minimally invasive approach when undergoing spinal fusion surgery. Tschugg et al. [16] retrospectively examined 67 patients, of which 19 patients underwent MITLIF and 48 patients underwent OTLIF, where lumbar spondylodiscitis was the indication for surgery. The MITLIF group showed favorable results with less postoperative pain, less blood loss, and a similar complication rate to OTLIF. According to Tschugg et al., MITLIF could be used safely and effectively in select cases of spondylodiscitis, even with epidural abscess [16].

To consider MITLIF as an alternative to OTLIF, it is important to determine the costs to evaluate its cost-effectiveness. Parker et al. [50] showed that the total and indirect hospital costs were significantly lower for MITLIF. Another study conducted by Pelton and colleagues to compare the perioperative costs in patients with and without workers’ compensation who underwent MITLIF or OTLIF showed that the total hospital costs were significantly lower for MITLIF with no difference between the workers’ compensation and nonworkers’ compensation patients [33].

## Conclusion

MITLIF is a novel surgical procedure developed to reduce approach-related morbidity associated with traditional OTLIF. To the best of our knowledge, ours is the largest literature review, with the largest number of included studies, comparing MITLIF and OTLIF. Our review showed that MITLIF provides good long-term clinical outcomes and is associated with less blood loss, and less postoperative pain with significantly less narcotic medications use, which lead to shorter LOS, earlier ambulation, and earlier return to work. Operative time remains a controversial parameter, with many studies showing longer operative time for MITLIF, some studies showing longer operative time for OTLIF, and still others showing no significant difference between the techniques. This heterogeneity is explained by the finding that our reviewed studies included MITLIF cases performed by surgeons during their initial learning curve, which required longer operative times. We suggest that MITLIF has a steep learning curve, but once adequate surgical experience is gained, the surgical time could be significantly reduced. This applies also to the complication rate, which is another controversial item. Complications associated with MITLIF included primarily screw misplacement and cage migration, attributed to the steep learning curve and the technical demands of MITLIF. Otherwise, we found no significant difference in complication rates between MITLIF and OTLIF.

Prolonged radiation exposure time is the main drawback of MITLIF, resulting primarily from the small operative field, lack of visualization of the bony landmarks, steep learning curve and, based on our experience, the prolonged fluoroscopy time needed to place the pedicle screws percutaneously. Therefore, long-term studies evaluating these parameters, namely operative time, complication rate, and radiation exposure time, are needed after adequate initial experience is gained. MITLIF may be associated with a lower risk of ASD; however, long-term studies are also needed to evaluate this parameter.

MITLIF is a safe alternative in obese patients and, in experienced hands, can also be used safely in select cases of spondylodiscitis even with epidural abscess. Economically, MITLIF is a cost-saving procedure associated with reduced hospital and social costs.

## Abbreviations

ASD: Adjacent segment disease
BMI: Body mass index
LOS: Length of hospital stay
MITLIF: Minimally invasive transforaminal lumbar interbody fusion
ODI: Oswestry Disability Index
OTLIF: Open transforaminal lumbar interbody fusion
TLIF: Transforaminal lumbar interbody fusion
VAS: Visual analogue scale
